# The mitochondrial genomes of five frog species of the Neotropical genus *Ischnocnema* (Anura: Brachycephaloidea: Brachycephalidae)

**DOI:** 10.1080/23802359.2018.1501312

**Published:** 2018-08-17

**Authors:** Pedro P. G. Taucce, Clarissa Canedo, Célio F. B. Haddad, Alan R. Lemmon, Emily Moriarty Lemmon, Miguel Vences, Mariana Lyra

**Affiliations:** a Department of Zoology and Aquaculture Center (CAUNESP), Biosciences Institute, São Paulo State University – UNESP, Rio Claro, Brazil;; b Department of Zoology, Instituto de Biologia Roberto Alcântara Gomes, Rio de Janeiro State University – UERJ, Rio de Janeiro, Brazil;; c Department of Scientific Computing, Florida State University, Tallahassee, FL, USA;; d Department of Biological Science, Florida State University, Tallahasse, FL, USA;; e Zoological Institute Technical University of Braunschweig, Braunschweig, Germany

**Keywords:** Amphibia, Brazil, *Ischnocnema guentheri* series, mitogenomes, Terrarana

## Abstract

We report the mitogenomes for five species of the *Ischnocnema guentheri* series, being the first described for this genus of brachycephalid frogs. We assembled mitogenomes from anchored hybrid enrichment data and recovered the 13 protein-coding genes, 22 tRNA genes, and two rRNA genes for all species. The general structure agrees with most previously sequenced neobatrachians, with two exceptions: the origin of replication of L-strand (O_L_) was found between tRNA-A and tRNA-N, and the position of tRNA-L and tRNA-T, which are dispersed in the control region. We provide a phylogenetic tree with outgroups, which is consistent with previous phylogenetic hypotheses.

The Neotropical genus *Ischnocnema* (Reinhardt and Lütken 1862) comprises 37 species (Frost 2018) of leaf-litter dwelling frogs divided into five species series and three species unassigned to any series (Taucce at al. 2018). Within this genus, the *I. guentheri* series comprises 10 species distributed all over the southern and central portions of the Atlantic Forest, throughout seven Brazilian states and the Argentinean province of Misiones (Frost 2018). The series has a challenging taxonomy, with notable intra and inter-specific morphological variation (Heyer 1984), and some of its members may actually represent complexes of morphologically cryptic species (Gehara et al. 2013). Herein, we provide complete or nearly complete metagenome sequences for half (five) of the currently recognized species of the *I. guentheri* series, assembled from anchored hybrid enrichment data (Lemmon et al. 2012): *I. erythromera* (Heyer 1984), *I. guentheri* (Steindachner 1864), *I. nasuta* (Lutz 1925), *I. oea* (Heyer 1984) (one specimen each), and *I. henselii* (Peters 1870) (five specimens). Voucher specimens and tissue samples are housed in the CFBH or LGE collections (acronyms follow Sabaj 2016).

We extracted total DNA from ethanol-preserved muscle or liver tissues using the DNeasy Qiagen^®^ kit following manufacturer’s protocols. DNA was eluted to a volume of 100 μl and quantified using a Qubit fluorometer dsDNA BR Assay Kit (Thermo Fisher Scientific Inc., Waltham, MA). Extractions were sent to the Center for Anchored Phylogenomics (Tallahassee, FL) to be sequenced with a method for anchored hybrid enrichment analysis (Lemmon et al. 2012). Samples were pooled after indexing and hybrid enrichments were performed with probes designed for anchored loci from amphibians (Barrow et al. 2018; Heinicke et al. 2018). Mitochondrial sequences were recovered as bycatch during the process of hybrid enrichment. Sequencing was carried out on an Illumina HiSeq2500 sequencer. For mitochondrial genomes assemblies, each lane of raw sequence reads was first concatenated per sample and quality-trimmed using Trimmomatic (Bolger et al. 2014). Then, we used MITObim v1.9 (Hahn et al. 2013) using as reference the mitogenome of *Eleutherodactylus atkinsi* (GenBank number: JX564864) to assemble the mitogenome of *Ischnocnema oea*. Next, we checked the quality and coverage of this new mitogenome and used it to assemble the remaining specimens of *Ischnocnema*. Assemblies were checked for quality by mapping the mitochondrial reads recovered by MITObim to the final fasta file with Geneious R11 (Kearse et al. 2012). We also used Geneious R11 to test for mitogenome circularity and completeness using the ‘De novo assemble’ tool. Regions with low coverage (less than eight reads) were manually edited to unknown nucleotides (‘N’). The preliminary annotation of final mitochondrial genomes was carried out by MITOS 2 (Bernt, Donath, Jühling, et al. 2013), available online at http://mitos2.bioinf.uni-leipzig.de/index.py, and verified by alignment with *Eleutherodactylus atkinsi*. The protein-coding regions were checked to confirm no indels or stop codons were present. The new mitogenomes have been deposited in GenBank under accession numbers MH492729–MH492737.

We recovered the typical 13 protein-coding genes, two rRNA genes and all 22 tRNA genes for all specimens, except *I. guentheri*. The mitogenome sequences of *I. nasuta*, one specimen of *I. henselii* and *I. guentheri* are complete and circular, but we could not annotate the tRNA-T in *I. guentheri*, probably because it is in a low coverage region. Other specimens are incomplete only in the control region (Supplementary Table S1; available at https://figshare.com/s/1537a00d5bf67288030e). Generally, the gene order observed agrees with most previously sequenced neobatrachian frogs (Zhang et al. 2013), with two main exceptions: the position of the putative origin of replication of the L-strand (O_L_), which we found between tRNA-A and tRNA-N (cluster WA(O_L_)NCY) and not between tRNA-N and tRNA-C (cluster WAN(O_L_)CY); and the organization of the LTPF cluster in the 5′ of the 12S rRNA gene, which differs in the *Ischnocnema* by the tRNA-L and tRNA-T being located within the control region (see Supplementary Figure S1). We also found a new arrangement for the cluster LTPF in *I. erythromera*, in which the tRNA arrangement is PLTF. These tRNAs clusters are known to be a hotspot of gene rearrangement in amphibians (San Mauro et al. 2006; Bernt, Braband, et al. 2013; Zhang et al. 2013) and the rearrangements in these regions are normally interpreted as a result of replication errors near the replication origins O-L and O-H (Kurabayashi and Sumida 2013).

**Table 1.** GenBank accession numbers, collection numbers, local of collection, and geospatial coordinates of specimens of the *Ischnocnema, Gastrotheca, Pristimantis,* and *Eleutherodactylus* species used in this study.

Species	GB accession number	Voucher	Local of collection	Coordinates (decimal degrees)
*I. erythromera*	MH492735	CFBH 40985	Teresópolis, RJ, Brazil	–22.45386, –42.99235
*I. guentheri*	MH492737	CFBH 27440	Rio de Janeiro, RJ, Brazil	–22.96192, –43.28912
*I. henselii*	MH492729	CFBH 41854	Bertioga, SP, Brazil	–23.73123, –46.17280
*I. henselii*	MH492732	CFBH 35837	Miracatu, SP, Brazil	–24.28223, –47.46796
*I. henselii*	MH492733	CFBH 27554	São Bonifácio, SC, Brazil	–27.87721, –48.94057
*I. henselii*	MH492731	CFBH 39280	São Francisco do Sul, SC, Brazil	–26.22797, –48.68011
*I. henselii*	MH492730	LGE 10099	San Pedro, Misiones, Argentina	–26.90000, –53.86667
*I. nasuta*	MH492734	CFBH 40981	Nova Friburgo, RJ, Brazil	–22.28923, –42.67095
*I. oea*	MH492736	CFBH 40987	Santa Teresa, ES, Brazil	–19.90706, –40.54034
*G. pseustes*	JX564866	TNHC 62492	Tixán, Chimborazo, Ecuador	–
*P. thymelensis*	JX564889	TNHC-GDC 14370	–	–
*P. fenestratus*	KT221610	–	Carolina, MA, Brazil	–
*E. atkinsi*	JX564864	MVZ 241209	Isla de la Juventud, Cuba	21.65495, –82.75922

For phylogenetic inference, *Ischnocnema* sequences were aligned to publish complete or near complete genomes of four outgroups (Table 1) using the software MAFFT v.7 (Katoh and Standley 2013). To avoid ambiguous alignments, we used only protein-coding and rRNA genes in the analyses. Search for the best partition scheme and best fitting nuclear substitution models was performed with PartitionFinder 2.1.1 (Lanfear et al. 2017). Phylogenetic analyses were performed under Bayesian inference (BI), maximum likelihood (ML), and maximum parsimony (MP) optimality criteria with the software MrBayes 3.2.6 (Ronquist et al. 2012), RAxML 8.2.11 (Stamatakis 2014), and TNT 1.5 (Goloboff and Catalano 2016), respectively. The best partition scheme with respective best-fitting substitution models and details on each phylogenetic analysis are given in Supplemental Online Material.

The three phylogenetic analyses are congruent and show all *Ischnocnema* species as a fully supported clade which is placed with high support as sister group to *Eleutherodactylus atkinsi* (Figure 1). The two species of *Pristimantis* (Jiménez de la Espada 1870) also appear as a fully supported clade. *Ischnocnema erythromera* is the sister species of all other *Ischnocnema* in our tree and *I. guentheri* and *I. henselii*, as well as *I. nasuta* and *I. oea*, appear, respectively, as sister species. The only clade in our phylogeny that did not receive strong support was *I. nasuta *+ *I. oea,* but only in the ML analysis (56% of bootstrap replicates). These results are congruent with the previous phylogenetic hypothesis encompassing all these *Ischnocnema* species based on a selection of mitochondrial and nuclear genes (Canedo and Haddad 2012 Taucce et al. 2018). The mitogenomes assembled here provide important information regarding the relationships within the *I. guentheri* species series and their genomic evolution.

**Figure 1.**
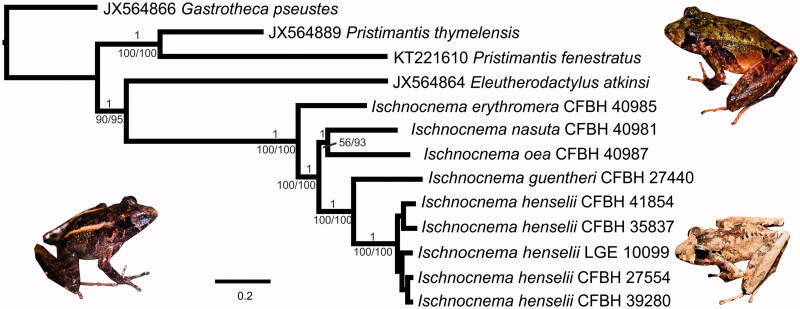
The 50% majority rule consensus tree from Bayesian inference analysis of mitogenomic sequences of Ischnocnema and four outgroups. Numbers above branches are posterior probabilities and numbers below branches are maximum likelihood bootstrap replicates (left) and maximum parsimony jackknife replicates (right). No support below species level is shown. Pictures show *Ischnocnema nasuta* (left), *I. erythromera* (above, right), and *I. henselii* (below, right).

## References

[CIT0029] Barrow L. N., Lemmon A. R., Moriarty Lemmon E. 2018 Targeted sampling and target capture: Assessing phylogeographic concordance with genome-wide data. Syst. Biol. Advance Access Syy021. (Accessed 21 March 2018). 10.1093/sysbio/syy021 30339251

[CIT0001] Bernt M, Donath A, Jühling F, Externbrink F, Florentz C, Fritzsch G, Pütz J, Middendorf M, Stadler PF 2013 MITOS: improved *de novo* metazoan mitochondrial genome annotation. Mol Phylogenet Evol. 69:313–319. 22982435 10.1016/j.ympev.2012.08.023

[CIT0002] Bernt M, Braband A, Schierwater B, Stadler PF 2013 Genetic aspects of mitochondrial genome evolution. Mol Phylogenet Evol. 69:328–338. 23142697 10.1016/j.ympev.2012.10.020

[CIT0003] Bolger AM, Lohse M, Usadel B 2014 Trimmomatic: a flexible trimmer for Illumina sequence data. Bioinformatics. 30:2114–2120. 24695404 10.1093/bioinformatics/btu170 PMC4103590

[CIT0004] Canedo C, Haddad CFB 2012 Phylogenetic relationships within anuran clade Terrarana, with emphasis on the placement of Brazilian Atlantic rainforest frogs genus *Ischnocnema* (Anura: Brachycephalidae). Mol Phylogenet Evol. 65:610–620. 22842090 10.1016/j.ympev.2012.07.016

[CIT0005] Frost DR 2018 Amphibian species of the world: an online reference. New York, USA: American Museum of Natural History.

[CIT0006] Gehara M, Canedo C, Haddad CFB, Vences M 2013 From widespread to microendemic: molecular and acoustic analyses show that *Ischnocnema guentheri* (Amphibia: Brachycephalidae) is endemic to Rio de Janeiro, Brazil. Conserv Genet. 14:973–982.

[CIT0007] Goloboff PA, Catalano SA 2016 TNT version 1.5, including a full implementation of phylogenetic morphometrics. Cladistics. 32:221–238. 10.1111/cla.12160 34727670

[CIT0008] Hahn C, Bachmann L, Chevreux B 2013 Reconstructing mitochondrial genomes directly from genomic next-generation sequencing reads—a baiting and iterative mapping approach. Nucl Acids Res. 41:e129. 23661685 10.1093/nar/gkt371 PMC3711436

[CIT0030] Heinicke M., Lemmon A.R., Moriarty Lemmon E., McGrath K., Blair Hedges S. 2018 Phylogenomic support for evolutionary relationships of New World direct-developing frogs (Anura: Terraranae). Mol. Phylogenet. Evol. 118:145–155. 28963082 10.1016/j.ympev.2017.09.021

[CIT0009] Heyer WR 1984 Variation, systematics, and zoogeography of *Eleutherodactylus guentheri* and closely related species (Amphibia: Anura: Leptodactylidae). Smith Contr Zool. 402:1.

[CIT0010] Jiménez de la Espada M 1870 Fauna neotropicalis species quaedam nondum cognitae. J Sci Math Phys Nat. 3:57–65.

[CIT0011] Katoh K, Standley DM 2013 MAFFT multiple sequence alignment software version 7: improvements in performance and usability. Mol Biol Evol. 30:772–780. 23329690 10.1093/molbev/mst010 PMC3603318

[CIT0012] Kearse M, Moir R, Wilson A, Stones-Havas S, Cheung M, Sturrock S, Buxton S, Cooper A, Markowitz S, Duran C, et al. 2012 Geneious Basic: an integrated and extendable desktop software platform for the organization and analysis of sequence data. Bioinformatics. 28:1647–1649. 22543367 10.1093/bioinformatics/bts199 PMC3371832

[CIT0013] Kurabayashi A, Sumida M 2013 Afrobatrachian mitochondrial genomes: genome reorganization, gene rearrangement mechanisms, and evolutionary trends of duplicated and rearranged genes. BMC Genomics. 14:633. 24053406 10.1186/1471-2164-14-633 PMC3852066

[CIT0014] Lanfear R, Frandsen PB, Wright AM, Senfeld T, Calcott B 2017 Partitionfinder 2: new methods for selecting partitioned models of evolution for molecular and morphological phylogenetic analyses. Mol Biol Evol. 34:772–773. 28013191 10.1093/molbev/msw260

[CIT0015] Lemmon AR, Emme SA, Lemmon EM 2012 Anchored hybrid enrichment for massively high-throughput phylogenomics. Syst Biol. 61:727–744. 22605266 10.1093/sysbio/sys049

[CIT0016] Lutz A 1925 Batraciens du Brésil. Comptes Rendus Mémoires Hebd des Séances la Société Biol des Ses Fil. 22:211–214.

[CIT0017] Padial JM, Grant T, Frost DR 2014 Molecular systematics of terraranas (Anura: Brachycephaloidea) with an assessment of the effects of alignment and optimality criteria. Zootaxa. 3825:1. 24989881 10.11646/zootaxa.3825.1.1

[CIT0018] Peters WCH 1870 Über neue Amphien (Hemidactylus, Urosaura, Tropdolepisma, Geophis, Uriechis, Scaphiophis, Hoplocephalus, Rana, Entomoglossus, Cystignathus, Hylodes, Arthroleptis, Phyllobates, Cophomantis) des Königlich Zoologisch Museum. Monatsberichte der Königlichen Preuss Akad des Wissenschaften Zu Berlin. 1870:641–652.

[CIT0019] Prum RO, Berv JS, Dornburg A, Field DJ, Townsend JP, Lemmon EM, Lemmon AR 2015 A comprehensive phylogeny of birds (Aves) using targeted next-generation DNA sequencing. Nature. 526:569–573. 26444237 10.1038/nature15697

[CIT0020] Reinhardt JT, Lütken CF 1862(1861). Bidrag til Kundskab om Brasiliens Padder og Krybdyr. Förste Afdeling: Padderne og Öglerne. Vidensk Meddelelser Fra Dansk Naturhistorisk Foren i Kjøbenhavn. 2:143–242.

[CIT0021] Ronquist F, Teslenko M, Van Der Mark P, Ayres DL, Darling A, Höhna S, Larget B, Liu L, Suchard MA, Huelsenbeck JP 2012 Mrbayes 3.2: efficient Bayesian phylogenetic inference and model choice across a large model space. Syst Biol. 61:539–542. 22357727 10.1093/sysbio/sys029 PMC3329765

[CIT0022] Ruane S, Raxworthy CJ, Lemmon AR, Lemmon EM, Burbrink FT 2015 Comparing species tree estimation with large anchored phylogenomic and small Sanger-sequenced molecular datasets: an empirical study on Malagasy pseudoxyrhophiine snakes. BMC Evol Biol. 15:1–14. 26459325 10.1186/s12862-015-0503-1 PMC4603904

[CIT0023] Sabaj MH 2016 Standard symbolic codes for institutional resource collections in herpetology and ichthyology: an online reference. Version 6.5 [accessed 2016 Aug 16]. http://www.asih.org/, American Society of Ichthyologists and Herpetologists 5:802–832. http://www.asih.org/resources, http://www.webcitation.org/6lkBdh0EO [accessed 2016 Nov 3].

[CIT0024] San Mauro D, Gower DJ, Zardoya R, Wilkinson M 2006 A hotspot of gene order rearrangement by tandem duplication and random loss in the vertebrate mitochondrial genome. Mol Biol Evol. 23:227–234. 16177229 10.1093/molbev/msj025

[CIT0025] Stamatakis A 2014 RAxML version 8: a tool for phylogenetic analysis and post-analysis of large phylogenies. Bioinformatics. 30:1312–1313. 24451623 10.1093/bioinformatics/btu033 PMC3998144

[CIT0026] Steindachner F 1864 Batrachologische Mittheilungen. Verhandlungen des Zool. Vereins Wien. 14:239–288.

[CIT0031] Taucce PPG, Canedo C, Parreiras JS, Drummond LO, Nogueira-Costa P, Haddad CFB 2018 Molecular phylogeny of Ischnocnema (Anura: Brachycephalidae) with the redefinition of its series and the desctiption of two new species. Molecular Phylogenetics and Evolution. doi:10.1016/j.ympev.2018.06.042 30146039

[CIT0027] Tucker DB, Colli GR, Giugliano LG, Hedges SB, Hendry CR, Lemmon EM, Lemmon AR, Sites JW, Pyron RA 2016 Methodological congruence in phylogenomic analyses with morphological support for teiid lizards (Sauria: Teiidae). Mol Phylogenet Evol. 103:75–84. 27395779 10.1016/j.ympev.2016.07.002

[CIT0028] Zhang P, Liang D, Mao RL, Hillis DM, Wake DB, Cannatella DC 2013 Efficient sequencing of anuran mtDNAs and a mitogenomic exploration of the phylogeny and evolution of frogs. Mol Biol Evol. 30:1899–1915. 23666244 10.1093/molbev/mst091

